# Effects of Dietary Choline Levels During Pregnancy on Reproductive Performance, Plasma Metabolome and Gut Microbiota of Sows

**DOI:** 10.3389/fvets.2021.771228

**Published:** 2022-01-24

**Authors:** Wei Zhong, Liang Hu, Yang Zhao, Zhen Li, Yong Zhuo, Xuemei Jiang, Jian Li, Xilun Zhao, Lianqiang Che, Bin Feng, Yan Lin, Shengyu Xu, Zhengfeng Fang, De Wu

**Affiliations:** ^1^Key Laboratory for Animal Disease-Resistance Nutrition of the Ministry of Education of China, Animal Nutrition Institute, Sichuan Agricultural University, Chengdu, China; ^2^College of Food Science, Sichuan Agricultural University, Ya'an, China

**Keywords:** gestation sows, choline, reproductive performance, microbiota, metabolomics

## Abstract

This study investigated the effects of dietary choline levels during gestation on reproductive performance of sows. In addition, the plasma metabolome and gut microbiota of sows was studied. A total of 260 multiparous sows were allocated to five dietary treatment groups with increasing choline concentrations (1,050, 1,450, 1,850, 2,250, and 2,650 mg/kg) in a randomized complete block design. The sows were fed experimental diets from breeding until farrowing and a common lactating diet during lactation. The results showed that the backfat (BF) gain of sows during gestation, individual birth weight for total piglets born, piglets born alive, average piglet weight at weaning increased linearly (*P* < 0.05), whereas the within-litter birth weight variation coefficient (CV) of piglets born alive and suckling piglet mortality decreased linearly (*P* < 0.05) as dietary choline level increased. A quadratic effect of dietary choline level was observed for the average daily feed intake (ADFI) of sows during lactation (*P* < 0.05). ADFI was maximized when the dietary choline concentration reached 1,910 mg/kg. Plasma H_2_O_2_ concentration at day 30 of gestation in the 1,050 mg/kg group was greater than that in the 1,850 and 2,650 mg/kg groups (*P* < 0.05). Plasma metabolomics identified 46 metabolites among the three groups. Specifically, plasma concentrations of trimethylamine-N-oxide (TMAO), dopamine, and L-proline increased while 1-methylhistidine concentration decreased as dietary choline levels increased. In addition, bacterial observed species and richness (Chao 1 and ACE) at day 110 of gestation decreased as dietary choline levels increased (*P* < 0.05). For the gut microbiota composition, the enhanced dietary choline level decreased the abundance of phylum *Proteobacteria* (*P* < 0.05) and increased the abundance of phylum *Actinobacteria* (*P* < 0.05) at day 30 of gestation. Compared with the 1,050 mg/kg group, the abundance of genus *Terrisporobacter* was less in the 1,850 mg/kg group, and genera *Bacillus* and *Cellulomonas* were greater in the 2,650 mg/kg group. In summary, increasing dietary choline levels improved the birth weight, uniformity of neonatal piglets and litter performance during lactation. This may be associated with better antioxidant capability, metabolic status, and gut microbiota of sows during gestation.

## Introduction

Over the past few decades, along with the selection of hyperprolific genotypes of sows, the individual piglet weight at birth and litter performance during lactation has decreased with increased litter size. This suggests that the nutritional supply for gestating sow needs to be re-evaluated (1, 2). Considering this finding, numerous studies have focused on the effects of macronutrients (e.g., energy and amino acids) intake during gestation on the reproductive performance of sows during the last few decades (3, 4). Several studies have shown that the increased intake of nutrients during gestation has a positive effect on piglet birth weight (5, 6). However, little research has been conducted on the effects of micronutrients, such as vitamins and minerals, on the reproductive performance of gestation sows and their litter performance.

Choline, as a water-soluble nutrient, is a dietary component essential for the normal function of all cells and participates in vital biological progress during fetal development (7). Choline can be synthesized endogenously, but its synthesis is not sufficient for optimal growth and health (8). It has been demonstrated that choline deficiency contributes to the development of various diseases in animals and humans, such as liver dysfunction and neurodegenerative diseases (9, 10). In addition, pregnancy is associated with a higher demand for choline due to accelerated one-carbon metabolism and the formation of new membranes as cells undergo division (11).

Due to limited research data, the recommendation of choline level, which was maintained at 1,250 mg/kg for optimal reproductive performance in the nutrient requirements of swine published by National Research Council (NRC, 2012), was originally presented by the NRC (1979). Over the past several decades, however, great advances in genetic selection, nutrition, management, and disease control have been incorporated into the pig industry, and modern hyperprolific sows farrow much more piglets than sows in 1970s (12). Hence, the choline recommendation proposed by NRC (2012) might need to be reappraised to achieve optimal reproductive performance for modern hyperprolific sows.

Therefore, we hypothesized that increasing maternal dietary choline levels during gestation could improve fetal growth and development. Accordingly, in the current study, we investigated the effects of different levels of maternal dietary choline intake on reproductive performance and litter growth. Specifically, we determined the plasma biochemical parameters, metabolomics, and gut microbiota of sows during gestation.

## Materials and Methods

The present experiment complied with the Animal Care and Use committee of Sichuan Agricultural University, and followed the current laws of animal protection (Ethic Approval Code: SCAUAC201408-3).

### Animals, Diets and Feeding

A total of 260 multiparous sows (Large White) with a parity of 2–8 were allocated to five dietary choline levels (1,050, 1,450, 1,850, 2,250, and 2,650 mg/kg) in a randomized complete block design. The batch of sows which were bred in the same week was considered a block. The distribution of the parities was similar in all five treatment groups. Sows were fed the experimental diets from day 0 (first artificial insemination) of gestation until farrowing. The basal diet was based on corn and soybean meal containing 2.07 Mcal NE per kg, 13.0% CP, 0.58% SID Lys, and 1,050 mg/kg choline (Table 1). To achieve the four additional choline concentrations, choline chloride (50%) was supplemented at the expense of corn, and the five diets contained 0, 0.107, 0.214, 0.321, and 0.428% choline chloride, respectively. The choline intake of sows in the 1,050 mg/kg group was designed to meet the daily choline requirements of gestating sows recommended by NRC (2012). Choline chloride was purchased from Yixing Akzonobel Chemical Co., Ltd. (Yixing, Jiangsu Province, China), and the choline content in choline chloride is 74.6%. The actual choline concentration in the basal diet was measured by ion chromatography at Sichuan Willtest Technology Co., Ltd. (Chengdu, Sichuan Province, China). Sows were fed 2.6 kg/day during early pregnancy (days 1–35 of gestation), 2.4 kg/day during mid-pregnancy (days 36–89 of gestation), and 3.2 kg/day late pregnancy (days 90 of gestation to parturition). After farrowing, sows were fed a common lactation diet (2.45 Mcal NE per kg, 17.2% CP, and 0.90% SID Lys; Table 1) in accordance with NRC (2012), and were offered the diet three times per day (i.e., at 0800, 1200, and 1500 h). Throughout the experiment, all animals had free access to water. Piglets were not allowed to receive creep feed during lactation.

**Table 1:** Composition and nutrient levels of gestation and lactation diets (as fed basis).

**Ingredient**	**Basal gestation diet**	**Lactation diet**
Corn	535.98	602
Wheat bran	100	40
Soybean hull	80	–
Sugar beet pulp	50	25
Rice bran meal	100	–
Soybean meal (CP 43%)	95	230
Fish meal (CP 65%)	–	20
Glucose	–	20
Limestone	10	13
Dicalcium phosphate	12	12
Soybean oil	–	25
Salt	5	5
L-Lysine HCl (98.5%)	1.5	1.5
L-Threonine (98.5%)	0.8	–
L-Valine (98.5%)	–	0.5
Magnesium oxide	1.5	–
Sodium bicarbonate	2	–
Mineral premix^a^	5	5
Vitamin premix^b^	0.5	0.5
Choline chloride (50%)	0.72	1
Total	1,000	1,000
**Calculated nutrient levels (%)^c^**		
NE (Mcal/kg)	2.07	2.45
CP	13.02 (12.84)^d^	17.19
Ca	0.80 (0.82)^d^	0.95
Total P	0.68 (0.72)^d^	0.7
Available P	0.32	0.37
Total Lys	0.70 (0.68)^d^	1.00
SID Lys	0.58	0.90
SID Met	0.19	0.26
SID Thr	0.45	0.55
SID Trp	0.12	0.14
Choline (mg/kg)	1,057 (1,040)^d^	1,546

a *Mineral mixture supplied per kilogram of diets: Fe 120 mg; Cu 20 mg; Mn 60 mg; Zn 120 mg; Se 0.3 mg; I 0.5 mg*.

b *Vitamin mixture supplied per kilogram of diets: vitamin A 10000IU; vitamin D_3_ 2000IU; vitamin E 60 IU; vitamin K_3_ 5.0 mg; vitamin B_1_ 5.0 mg; vitamin B_2_ 10.0 mg; vitamin B_6_ 6.0 mg; vitamin B_12_ 50 μg; nicotinic acid 40 mg; d-pantothenic acid 20 mg; folic acid 2.0 mg; biotin 0.2 mg*.

c *Calculated chemical concentrations using values for feed ingredients from the Table of composition and nutritional value of Chinese feed (2017)*.

d *Data in brackets were analyzed values. To achieve the four additional choline concentrations, choline chloride (50%) was supplemented at the expense of corn and the five diets contained 0, 0.107, 0.214, 0.321 and 0.428% choline chloride, respectively*.

*CP, crude protein; NE, net energy; SID, standardized ileal digestible*.

### Recording and Sampling

The BF thickness of all sows was measured at 65 mm on the left side of the dorsal midline at the last rib on days 0 and 112 of gestation and at weaning using B-mode ultrasonography (LN-9300A, Liaoning Caresono Technology Co., Ltd, Dandong, Liaoning Province, China). After parturition, the total number of pigs born, born alive, mummified, and stillborn for each sow was recorded. The weight of the piglets was recorded individually at parturition and weaning. Cross-fostering was carried out within diet treatments to adjust litter size to 11 ± 1 piglets per sow within 24 h after parturition according to the number of effective teats of sows. After weaning, estrus detection was performed once a day and weaning-to-estrus interval (WEI) was recorded after estrus confirmation by standing heat in the presence of a boar.

Eight sows in the 1,050 mg/kg (L), 1,850 mg/kg (M) and 2,650 mg/kg (H) groups were randomly selected for sample collection. Fresh feces were collected directly by massaging the rectum of sows at days 30 and 110 of gestation. Subsequently, fecal samples were transported to the laboratory on dry ice and then stored at −80°C until analysis. A 10-ml blood sample was collected from the sows' ear veins at days 30, 90, and 110 of gestation after an overnight fasting period of 16 to 18 h. Plasma samples were obtained by centrifuging blood samples at 3,000 g for 15 min at 4°C after standing for 1 h at 4°C. The samples were immediately stored at −80°C for further analysis. The colostrum samples were collected within 1 h after the onset of farrowing and milk was collected on day 14 of lactation after oxytocin injection.

### Composition of Colostrum and Milk

Frozen samples were thawed at 4°C. 18 ml of each sample was used for milk composition analysis. Dry matter (DM), protein, fat, lactose, and solids-non-fat content were measured using a milk composition analyzer (Milkoscan 4000; Foss MilkoScan, Hillerød, Denmark).

### Assessment of Biochemical Parameters and Hormones in the Plasma

Plasma concentrations of urea, alanine aminotransferase (ALT), aspartate aminotransferase (AST), gamma-glutamyl transpeptidase (γ-GGT), immunoglobulin G (IgG), immunoglobulin M (IgM), total cholesterol (TC), total bile acid (TBA), and glucose (GLU) at days 30 and 110 of gestation were measured using an automatic biochemical analyzer (Model 7020, Hitachi, Tokyo, Japan) according to the corresponding commercial kits (Sichuan Maker Biotechnology Inc., <city>Chengdu </city>,China). Plasma concentrations of insulin-like growth factor 2 (IGF-2), homocysteine (HCY) and 17β-estradiol were assayed by ELISA using a commercial kit (R&D Systems Europe, Abingdon, UK), according to the manufacturer's instructions. Plasma acetylcholine and progesterone concentrations were determined using a commercial ELISA assay kit (Sigma-Aldrich, USA) according to the manufacturer's instructions.

### Measurement of Antioxidant Parameters, Lipid Peroxidation and Hydrogen Peroxide (H_2_O_2_)

Plasma at days 30 and 110 of gestation was used to determine the content of malondialdehyde (MDA), superoxide dismutase (SOD), total antioxidant capability (T-AOC) and hydrogen peroxide (H_2_O_2_) using commercial assay kits (Nanjing Jiancheng Institute, Jiangsu, China). The plasma concentration of MDA was quantified using the thiobarbituric acid method according to Che et al. (1). Plasma SOD and T-AOC concentrations were quantified as described by Hu et al. (13). Plasma H_2_O_2_ levels were measured according to the method described by Meng et al. (14). H_2_O_2_ bound to molybdenic acid to form a complex, which was measured at 405 nm. All plasma samples were measured in duplicate and the mean values were used for statistical analysis.

### Metabolomics Based on Ultra-High-Performance Liquid Chromatography Time-of-Flight/Mass Spectrometry

After pretreatment, plasma samples at day 90 of gestation were separated using an ultra-high-performance liquid chromatography (UHPLC) system (1290 Infinity II, Agilent Technologies, Santa Clara, CA, USA) incorporating an HILIC column (2.1 × 100 mm, 1.7 μm; Waters, Milford, MA). The samples were analyzed using a triple time-of-flight (TOF) 5600+ system (AB/SCIEX, Framingham, MA) equipped with an electrospray ionization source used in positive and negative ion modes. The pretreatment, extraction, and identification of serum samples were performed according to the procedure described by Hu et al. (15). The data were processed using the Xcalibur 2.3 data processing system. The retention time (Rt), mass to charge ratio (m/z), and fold change of each sample were entered into an Excel spreadsheet and then imported into SIMCA-P (version 13.0, Umetrics AB, Sweden) software for multivariate statistical analysis. Principal component analysis (PCA) was used to determine intra-group aggregation and inter-group separation tendencies, whereas Orthogonal partial least squares discriminant analysis (OPLS-DA) was performed to further determine inter-group differences. Significantly different metabolites were screened using variable importance in projection (VIP) scores (VIP > 1) obtained from the OPLS-DA model and *P* values (*P* < 0.05). Finally, the high-resolution mass spectrometer (MS) spectral data and MS/MS spectral data were matched with the HMDB (http://hmdb.ca/) and MassBank (http://massbank.jp) databases. Metabolite identification and Kyoto Encyclopedia of Genes and Genomes (KEGG) pathway analysis were performed according to the procedure described by Hu et al. (15).

### Analysis of the Gut Microbiota

The fecal samples were sent to a commercial company (Novogene, Beijing, China) for microbiota analysis. Microbial DNA was extracted from 0.25 g of fecal samples using the CTAB/SDS method. DNA concentration and purity were monitored on a 1% agarose gel. According to the concentration, DNA was diluted to 1 ng/μl using sterile water. The DNA was amplified using PCR with primers 515F (5′-GTGCCAGCMGCCGCGGTAA-3′) and 806R (5′- GGACTACNNGGGTATCTAAT-3′), which target the V4 region of the 16S rDNA gene. Sequencing was conducted on an Ion S5TM XL platform, which generated 400 bp/600 bp single-end reads. Bioinformatics analysis was conducted following a recent study (16).

### Statistical Analysis

All calculations and statistical analyses were performed using SAS software (SAS 9.4, Inst, Inc., Cary, NC, USA) with the individual sow as the experimental unit and dietary treatment as a fixed effect.

For reproductive and lactation performance data, the week of onset of the feed treatment was included as a random effect in the models. Polynomial contrasts were used to evaluate the linear and quadratic effects of the dose response of dietary choline levels. The percentage of pregnant sows, stillborn, mummified fetuses, and piglets which died during lactation were analyzed using the GLIMMIX procedure, and fitted with the assumption that the data exhibited a binomial distribution. The total number of piglets born, born alive piglets, born alive piglets weighing above 800 g, litter size after cross-fostering, litter size at weaning and WEI were analyzed using the GLIMMIX procedure with the Poisson distribution. Backfat at days 0 and 112 of gestation, litter weight and individual piglet weight at birth, litter weight and individual piglet weight after cross-fostering and at weaning, piglet average daily gain (ADG), sow ADFI, BF change from day 0 to day 112 of gestation and from day 112 of gestation to day 18 of lactation were continuous data and were analyzed using the MIXED procedure fitted assuming a normal distribution with DDFM = KR options included in the model. The UNIVARIATE procedure was used to test residuals for outliers. A residual analysis was conducted to check the model assumptions. Normality checks were carried out using PROC UNIVARIATE with NORMAL and PLOT options. The CV of within-litter piglet weight at birth, after cross-fostering and at weaning was analyzed using the GLIMMIX procedure, considering that the data exhibited a beta distribution, as all observed values were between 0 and 1. In the analysis of individual piglet birth weight and BF change during gestation and lactation, total piglets born, born alive, and BF at days 0 and 112 of gestation were used as covariate in the model respectively.

In the analysis of the plasma biochemical parameters, colostrum and milk composition, alpha diversity, and relative abundance data at the phylum and genus levels, the UNIVARIATE procedure was used to test the residuals for outliers first and then the data normality. The data which fit the normality distribution were analyzed using the MIXED procedure, and the data which did not fit the normality distribution were analyzed using the MIXED procedure after log10 transfer. Data which did not fit the normality distribution after log10 transfer were analyzed by non-parametric tests. An independent sample *t*-test was used for the plasma differential metabolite analysis.

The differences among the treatments were compared using a multiple comparison test, following the Tukey method. Data are presented as least squares means with pooled standard error of mean (SEM) unless otherwise specified. For all statistical analyses, significance was declared at *P* < 0.05, and the tendency at 0.05 < *P* ≤ 0.10.

## Results

### Reproductive Performance of Sows

As shown in Table 2, no differences were found among treatments at the onset of the experiment (*P* > 0.05) for parity and BF. No effect of dietary choline level during gestation was found on the pregnancy rate, total number of piglets born, piglets born alive, piglets weighing above 800 g, stillborn and mummified fetuses (*P* > 0.05). BF loss of sows during lactation was not affected by the choline levels in gestational diets (*P* > 0.05, Table 3). No differences among treatments were observed for the number of piglets weaned, average weight of piglets at weaning, ADG of piglets during lactation and WEI of the sows (*P* > 0.05, Table 3). Increasing dietary choline levels significantly increased the BF gain of sows during gestation (*P* = 0.041) and decreased the within-litter birth weight CV of piglets born alive (*P* = 0.048). Moreover, the BF gain of sows during gestation, individual birth weight for total piglets born, piglets born alive, and average piglet weight at weaning increased linearly as dietary choline level increased (*P* < 0.05; Tables 2, 3), whereas the within-litter birth weight CV of piglets born alive and suckling piglet mortality decreased linearly (*P* < 0.05) as dietary choline level increased. In addition, a quadratic effect of dietary choline level was observed for the ADFI of sows during lactation (*P* < 0.05; Table 3) and the choline level to maximize ADFI was calculated to be 1,910 mg/kg according to the quadratic equation.

**Table 2:** Effect of dietary choline levels during gestation on sow reproductive performance^1^.

**Item**	**Dietary choline level, mg/kg**					**SEM**	***P*** **-value**		
	**1,050**	**1,450**	**1,850**	**2,250**	**2,650**		**ANOVA**	**Linear**	**Quadratic**
Parity	4.37	4.29	4.25	4.23	4.29	0.276	1.000	0.816	0.789
Pregnancy rate, %	98.1	98.1	96.2	94.2	92.3	2.77	0.577	0.115	0.911
BF at day 0, mm	13.36	13.20	13.18	13.49	13.30	0.394	0.971	0.874	0.832
BF at day 112, mm	15.01	14.95	14.92	15.33	15.71	0.409	0.617	0.197	0.333
BF gain (day 0–112), mm^2^	1.64^b^	1.74^ab^	1.75^ab^	1.81^ab^	2.42^a^	0.196	0.041	0.014	0.158
Total piglets born	13.51	13.14	13.74	12.68	12.66	0.663	0.539	0.203	0.638
Born alive	12.18	12.05	12.32	11.57	11.51	0.639	0.747	0.264	0.655
Piglets weighing > 800 g	11.48	11.46	11.92	11.25	11.08	0.605	0.831	0.521	0.457
Stillborn, %	8.56	7.67	7.17	6.29	7.77	1.108	0.672	0.385	0.284
Mummified fetuses, %	1.31	0.77	1.09	1.07	1.05	0.45	0.922	0.930	0.653
**Total born**									
Litter birth weight, kg^3^	17.79	18.34	19.04	17.85	18.10	0.837	0.671	0.953	0.308
Piglet birth weight, kg^4^	1.34	1.41	1.39	1.42	1.45	0.032	0.115	0.037	0.643
Birth weight CV, %^3^	23.5	20.1	21.2	22.8	20.4	1.24	0.189	0.279	0.677
**Born alive**									
Litter birth weight, kg	16.50	17.01	17.81	16.85	16.83	0.808	0.671	0.803	0.236
Piglet birth weight, kg^4^	1.37	1.41	1.44	1.47	1.47	0.030	0.048	0.010	0.422
Birth weight CV, %	20.1	19.5	18.6	18.1	17.6	0.90	0.260	0.023	0.886

1 *N = 44–50 per group. Data are presented as least squares means with pooled SEM*.

2 *BF at day 0 of gestation was used as covariate for BF gain (day 0–day 112)*.

3 *Calculated considering the number of born alive + stillborn + mummifed fetuses*.

4 *Total piglets born and born alive were used as covariate for birth weight CV of total born and that of born alive respectively*.

a, b *Means within a row with different superscripts indicate significant differences (P < 0.05)*.

*BF, backfat*.

**Table 3:** Effect of dietary choline levels during gestation on lactation performance of sows^1^.

**Item**	**Dietary choline level, mg/kg**					**SEM**	***P*** **-value**		
	**1,050**	**1,450**	**1,850**	**2,250**	**2,650**		**ANOVA**	**Linear**	**Quadratic**
ADFI, kg	6.05	6.31	6.46	6.26	6.14	0.169	0.103	0.717	0.009
BF loss, mm^2^	1.15	0.92	1.01	1.01	1.06	0.237	1.000	0.968	0.863
**After cross-fostering**									
Litter size	10.68	10.80	10.73	10.56	10.74	0.542	0.996	0.952	0.989
Litter weight, kg	15.32	16.18	16.70	16.05	16.44	0.421	0.127	0.091	0.155
Average piglet weight, kg	1.42	1.49	1.55	1.52	1.53	0.039	0.064	0.012	0.114
**Weaned, day 18**									
Litter size	10.12	10.02	10.48	10.38	10.40	0.531	0.942	0.678	0.896
Litter weight, kg	55.38	55.93	59.82	58.81	59.12	1.590	0.143	0.034	0.358
Average piglet weight, kg	5.46	5.56	5.68	5.71	5.72	0.150	0.408	0.063	0.544
Piglet ADG, g	221.2	219.8	227.4	231.6	229.9	7.16	0.497	0.106	0.849
Piglet mortality (day 0–day 18), %	4.94^ab^	6.72^a^	2.18^b^	1.57^b^	2.85^ab^	1.05	0.001	0.003	0.212
WEI, d	5.88	6.08	5.27	5.19	6.10	0.426	0.368	0.720	0.175

1 *N=35–42 per group. Data are expressed as least squares means with pooled SEM*.

a, b *Means within a row with different superscripts indicate significant differences (P < 0.05)*.

2 *BF difference calculated between day 112 of gestation and day 18 of lactation. BF at day 112 of gestation was used as covariate*.

*ADFI, average daily feed intake; ADG, average daily gain; WEI, wean-to-estrus interval*.

### Milk Composition and Plasma Biochemical Profiles

No differences were found among the treatments for colostrum and milk composition of sows (*P* > 0.05; Table 4). Plasma urea concentration at day 30 of gestation tended to increase (*P* = 0.054; Table 5), whereas plasma AST concentration tended to decrease at day 110 of gestation (*P* = 0.075) as dietary choline levels increased.

**Table 4:** Effect of dietary choline levels during gestation on colostrum and milk composition of sows.

**Item**	**Dietary choline level, mg/kg**			**SEM**	***P*-value**
	**1,050**	**1,850**	**2,650**		
**Colostrum**					
Dry matter, %	25.65	26.60	25.32	0.813	0.513
Solids-non-fat, %	22.22	22.64	21.37	0.604	0.339
Lactose, %	2.97	3.14	2.96	0.143	0.621
Protein, %	15.59	15.93	14.74	0.698	0.482
Fat, %	3.76	4.24	4.35	0.338	0.441
**Milk**					
Dry matter, %	21.59	20.86	21.09	0.588	0.657
Solids-non-fat, %	14.34	14.37	14.27	0.211	0.941
Lactose, %	5.62	5.81	5.72	0.092	0.347
Protein, %	5.29	5.11	5.11	0.193	0.740
Fat, %	7.85	7.16	7.41	0.464	0.558

*Data are expressed as least squares means with pooled SEM, N = 8 per treatment*.

**Table 5:** Effect of dietary choline levels during gestation on plasma biochemical profiles of sows.

**Item**	**Dietary choline level, mg/kg**			**SEM**	***P*-value**
	**1,050**	**1,850**	**2,650**		
**Day 30**					
ALT, U/L	32.29	31.50	33.00	2.042	0.870
AST, U/L	18.50	23.14	19.33	1.770	0.186
γ-GGT, U/L	32.25	34.43	28.30	3.827	0.510
IgG, g/L	3.82	4.05	3.81	0.099	0.193
IgM, g/L	0.58	0.56	0.57	0.026	0.834
GLU, mmol/L	4.20	4.34	4.05	0.137	0.341
Urea, mmol/L	3.26	3.51	4.03	0.227	0.054
TC, mmol/L	1.51	1.49	1.51	0.120	0.996
TBA, umol/L	11.57	13.70	11.80	2.253	0.780
**Day 110**					
ALT, U/L	30.78	32.14	30.56	2.300	0.880
AST, U/L	31.00	25.75	24.56	4.749	0.075
γ-GGT, U/L	38.67	34.25	29.56	4.907	0.421
IgG, g/L	3.86	3.72	3.67	0.104	0.419
IgM, g/L	0.57	0.55	0.54	0.029	0.698
GLU, mmol/L	4.17	4.47	4.33	0.110	0.183
Urea, mmol/L	3.67	3.83	3.25	0.303	0.396
TC, mmol/L	1.32	1.47	1.19	0.089	0.114
TBA, umol/L	8.20	9.66	9.31	1.039	0.598

*Data are expressed as least squares means with pooled SEM, N = 8 per treatment*.

*GLU, glucose; ALT, alanine aminotransferase; AST, aspartate aminotransferase; γ-GGT, gamma-glutamyl transpeptidase; IgG, immunoglobulin G; IgM, immunoglobulin M; TC, total cholesterol; TBA, total bile acid*.

### Plasma Concentrations of Hormone, Antioxidant Related Parameters, Cytokines, Acetylcholine and HCY

**Figure 1:**
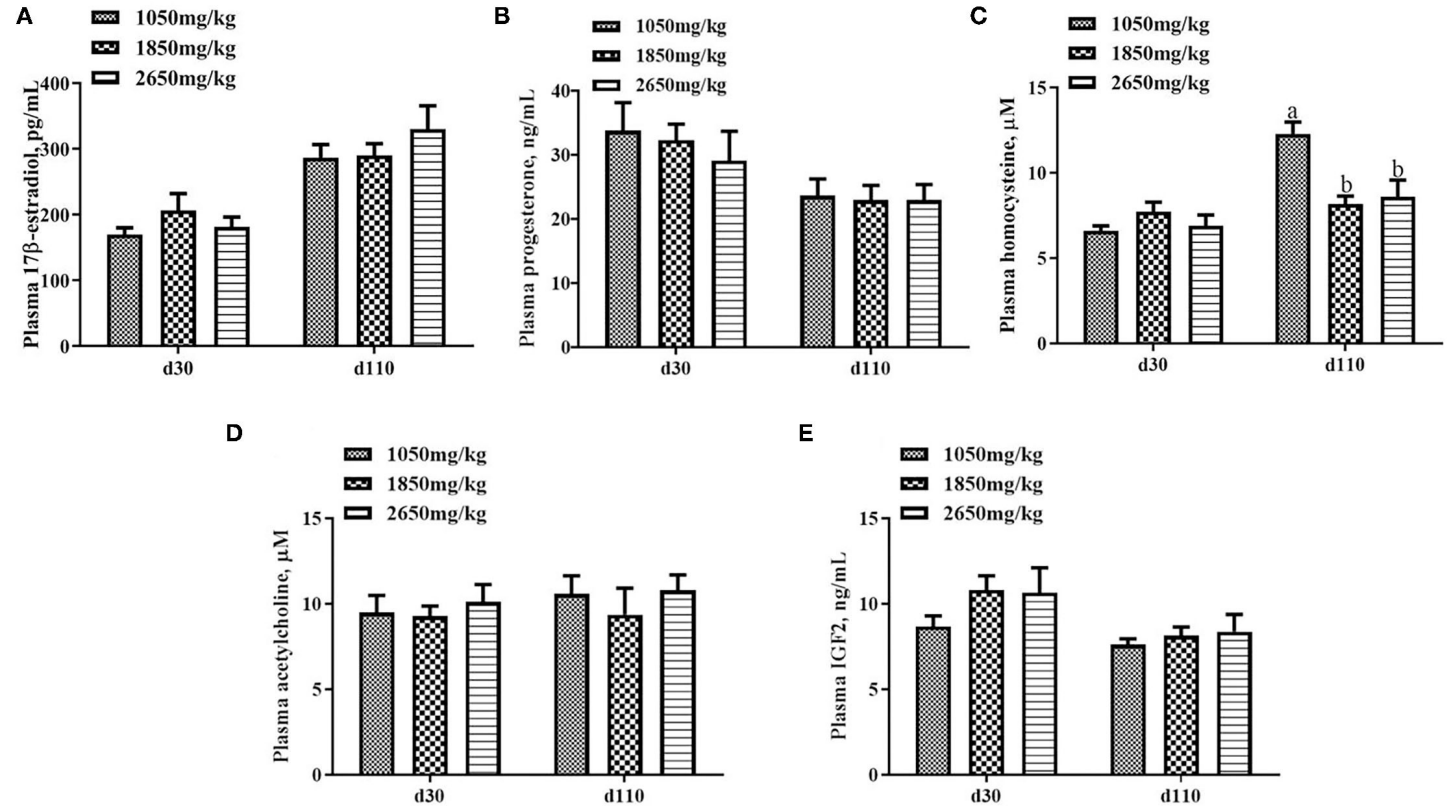
Effect of dietary choline levels during gestation on the plasma concentrations of 17β-estradiol **(A)** progesterone, **(B)** HCY, **(C)** acetylcholine, **(D)** IGF_2_, and **(E)** at days 30 and 110 of gestation. Data are expressed as mean values with their standard errors, *N* = 8 for each treatment. a, b Mean values with unlike letters were significantly different (*P* < 0.05).

**Figure 2:**
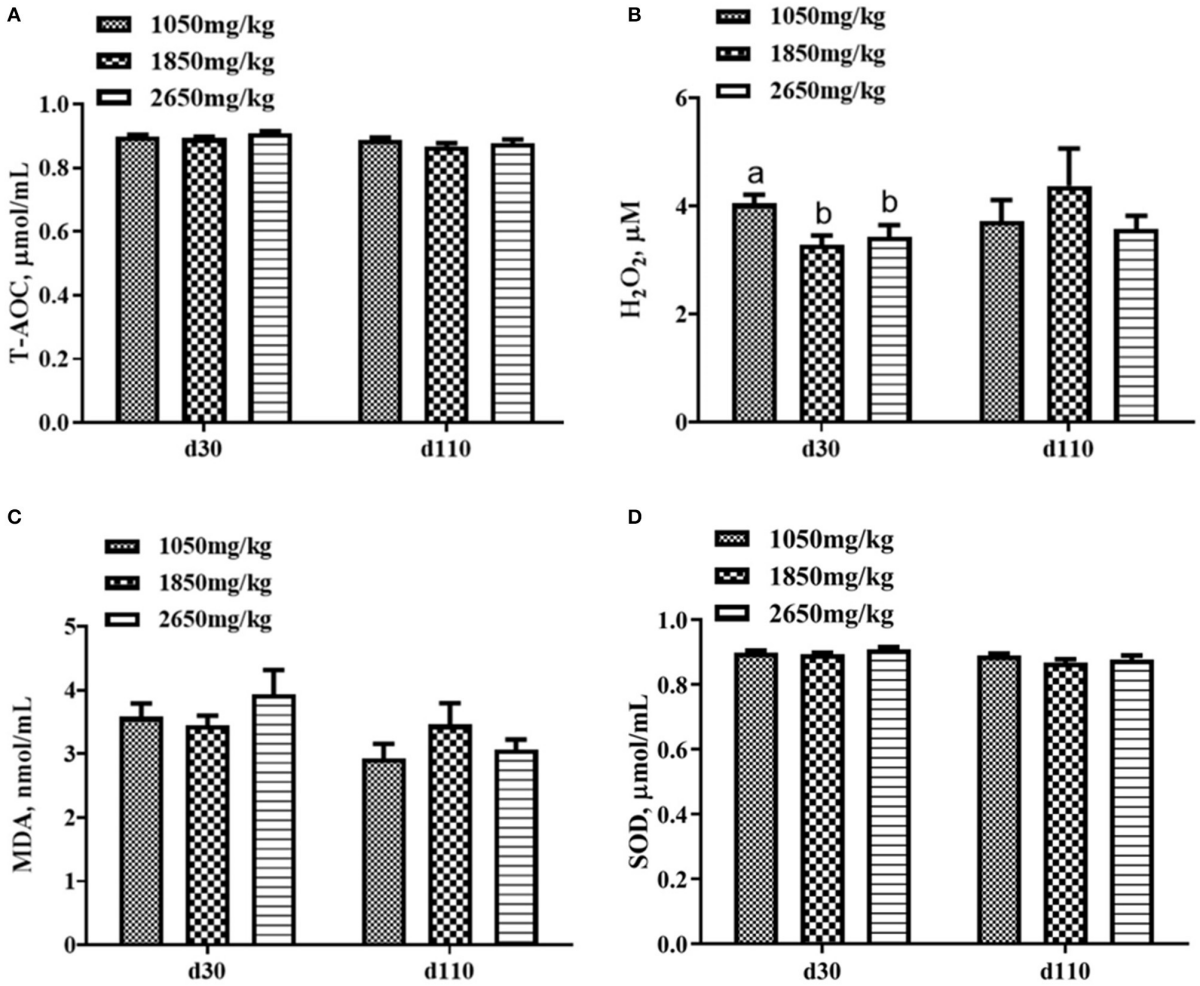
Effect of dietary choline levels during gestation on the plasma concentrations of T-AOC **(A)** H_2_O_2_, **(B)** MDA, **(C)** SOD, and **(D)** at days 30 and 110 of gestation. Data are expressed as mean values with their standard errors, *N* = 8 for each treatment. a, b Mean values with unlike letters were significantly different (*P* < 0.05).

**Figure 3:**
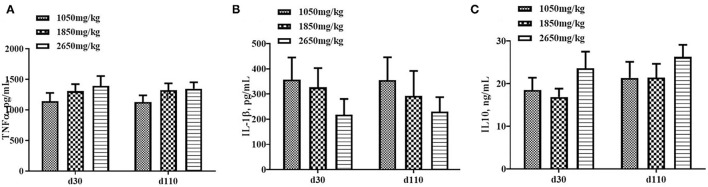
Effect of dietary choline levels during gestation on the plasma concentrations of TNFα **(A)** IL-1β, **(B)** IL10, and **(C)** at days 30 and 110 of gestation. Data are expressed as mean values with their standard errors, *N* = 8 for each treatment.

Plasma concentrations of HCY at day 110 (*P* < 0.05; Figure 1C) and H_2_O_2_ at day 30 of gestation (*P* < 0.05; Figure 2B) in the 1,050 mg/kg choline group were greater than those in the 1,850 and 2,650 mg/kg choline groups. No significant differences were found among the three groups in the plasma concentrations of 17β-estradiol, progesterone, acetylcholine, IGF_2_,T-AOC, MDA, SOD,TNFα,IL-1β and IL10 at days 30 and 110 of gestation (*P* > 0.05, Figures 1–3). In addition, there was no significant differences among the three groups for the plasma concentrations of HCY at day 30 (*P* > 0.05; Figure 1C) and H_2_O_2_ at day 110 of gestation (*P* > 0.05; Figure 2B).

### Plasma Metabolic Profiles

**Figure 4:**
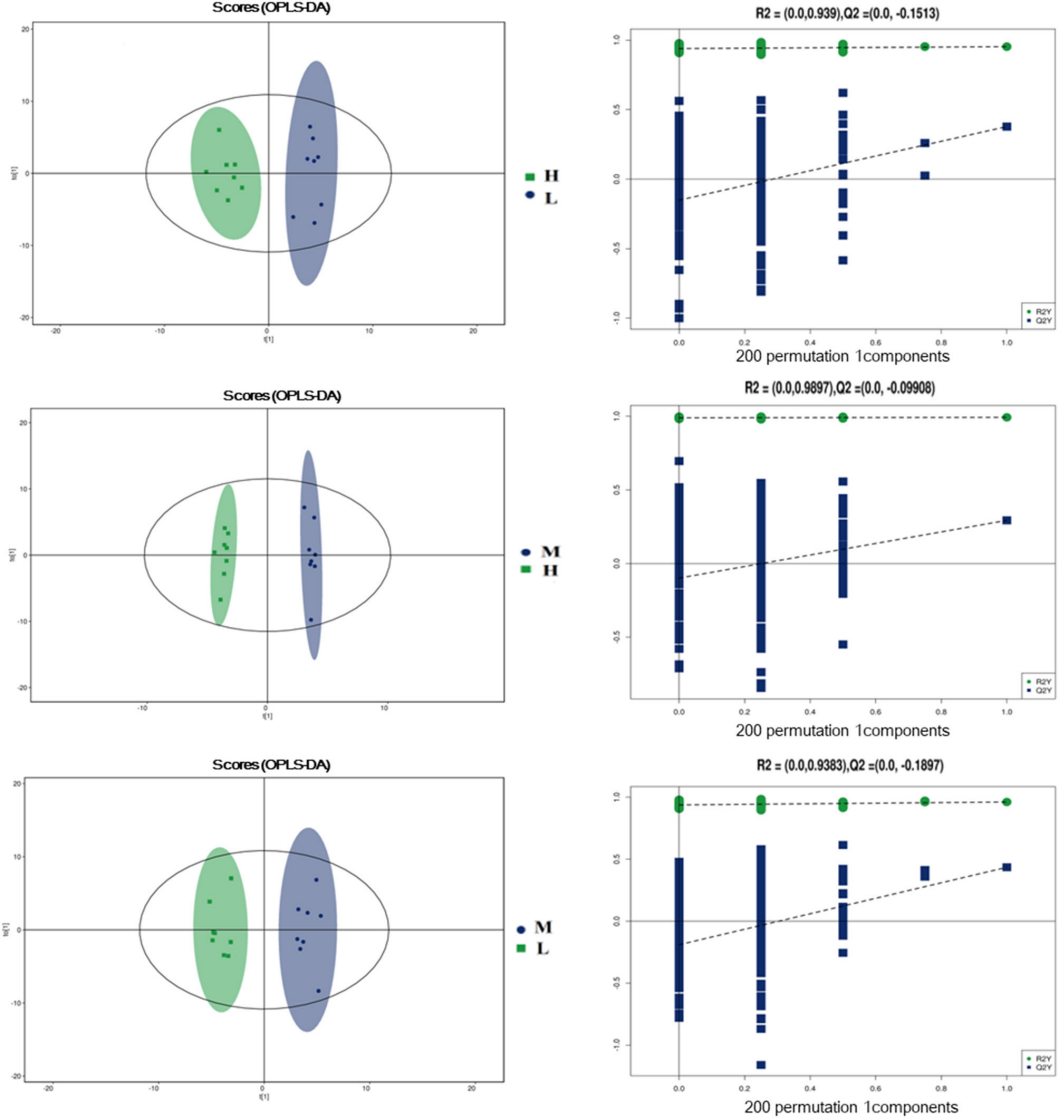
Orthogonal partial least squares discriminant analysis (OPLS-DA) score plots in positive electrospray ionization mode (ESI+) metabolomics profiles of plasma. L = 1,050 mg/kg choline group; M = 1,850 mg/kg choline group; H = 2,650 mg/kg choline group.

**Figure 5:**
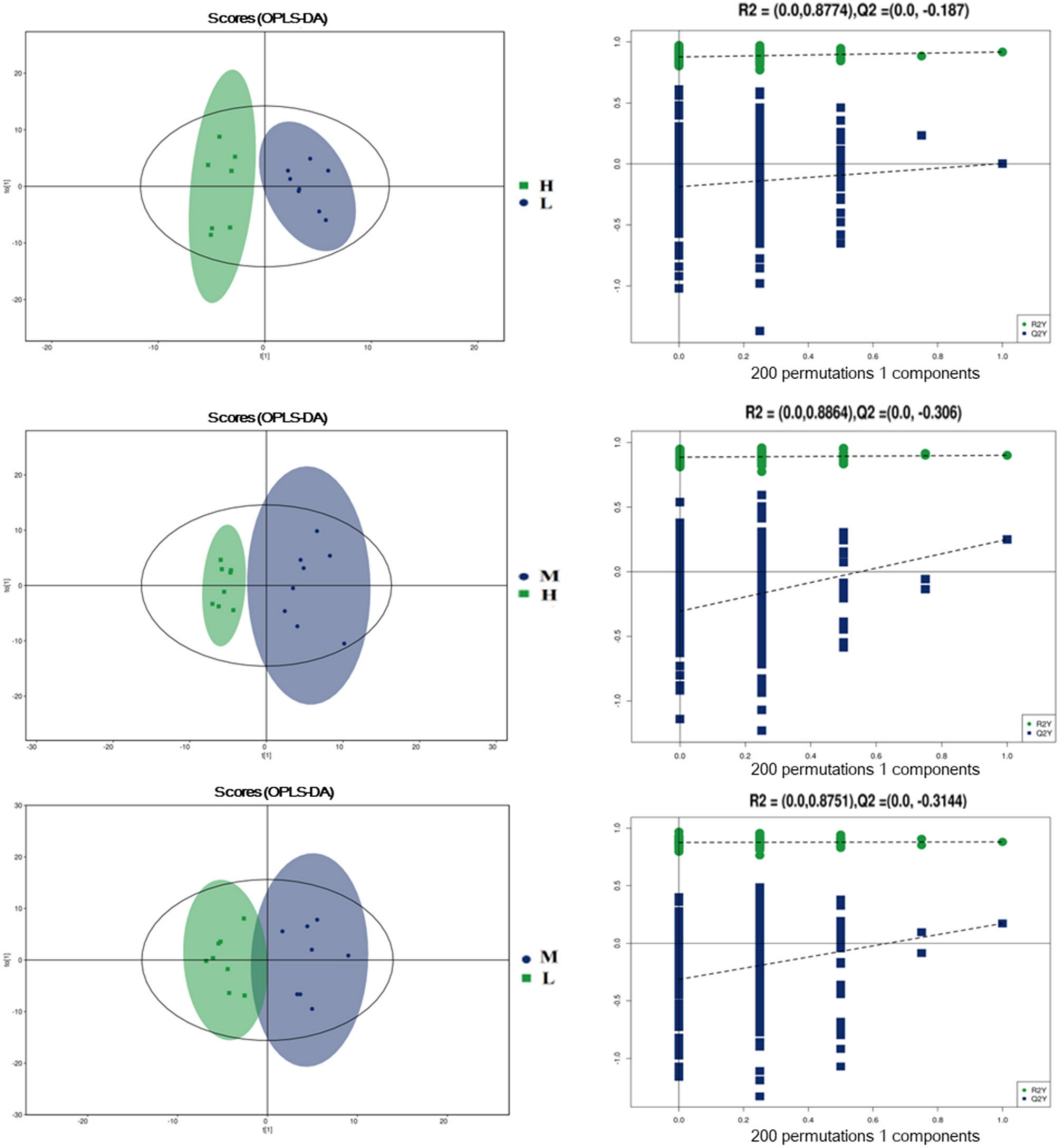
Orthogonal partial least squares discriminant analysis (OPLS-DA) score plots in negative electrospray ionization mode (ESI-) metabolomics profiles of plasma. L = 1,050 mg/kg choline group; M = 1,850 mg/kg choline group; H = 2,650 mg/kg choline group.

**Figure 6:**
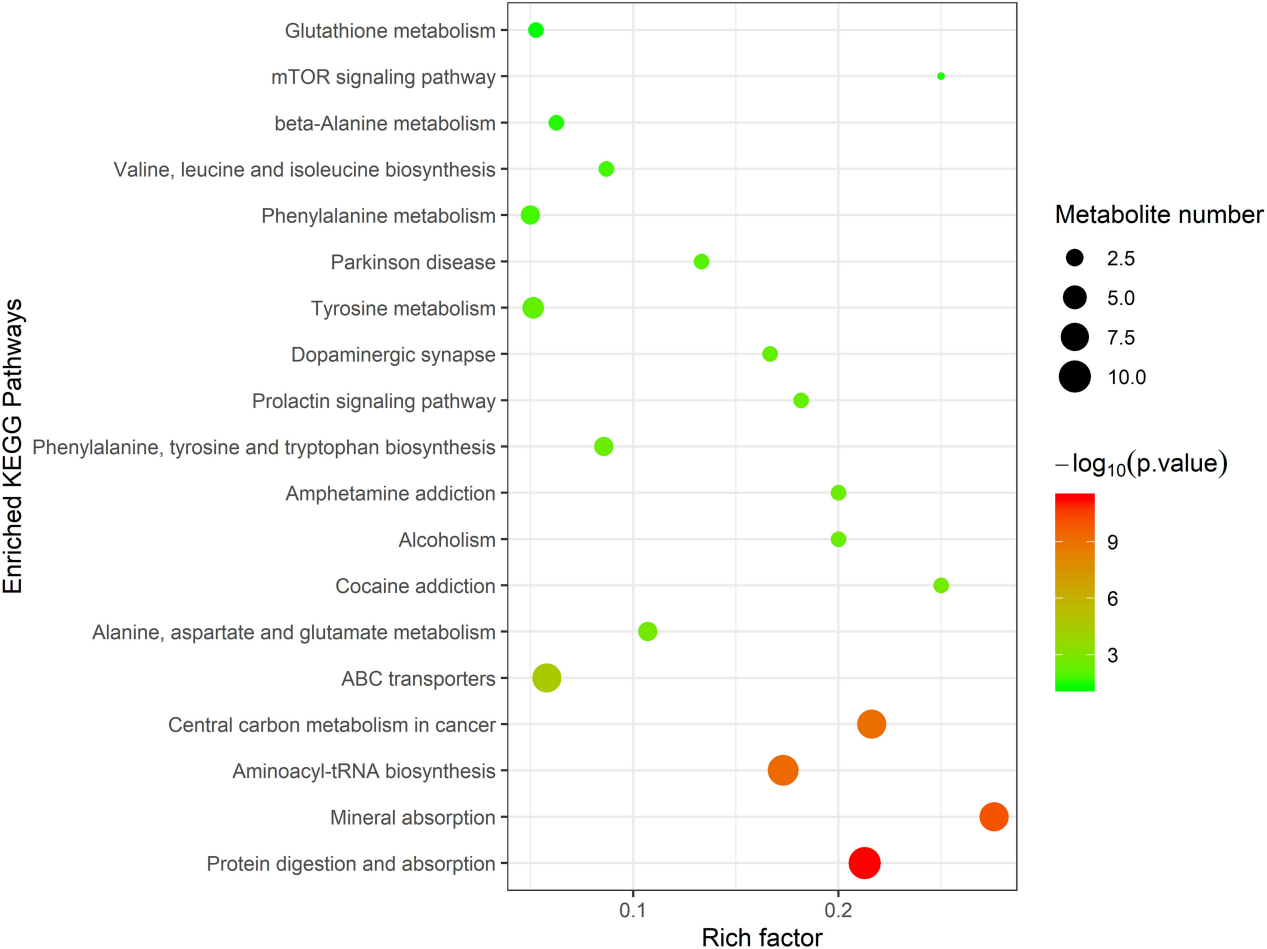
Topology analysis of metabolic pathways identified among 1,050 mg/kg choline group, 1,850 mg/kg choline group, and 2,650 mg/kg choline group. The X-axis represents the rich factor, and the Y-axis represents the pathway. Larger sizes and darker colors represent greater pathway enrichment and higher pathway impact values, respectively.

To further evaluate the differences in metabolic profiles induced by dietary choline intake, Ultra high pressure liquid chromatography - Quadrupole time of flight - Mass Spectrometry (UHPLC-QTOF-MS) was used to identify the differential metabolites. PCA and OPLS-DA were performed to visualize the Liquid Chromatograph-Mass Spectrometer (LC-MS) dataset and exhibit the differences and similarities among the samples. No significant difference among these groups in the PCA analysis (Supplementary Figures 1, 2). To further investigate the discrimination, OPLS-DA analyses were performed. The OPLS-DA score plots show separation among these groups in both positive (Figure 4) and negative (Figure 5) modes. To assess which compounds were responsible for the differences among these groups, the parameters of VIP > 1.0 and adjusted *P* < 0.10 were used as key lineages for separating the plasma compounds among these groups. Based on the high-resolution mass measurement of molecular ions and fragmentation ions, a total of 46 different plasma metabolites among the three groups were annotated and are listed in Supplementary Table 1. Pathway enrichment and pathway topology analysis were performed, which were based on high-quality KEGG metabolic pathways as the backend knowledgebase. As shown in Figure 6, these metabolites were involved in multiple biochemical pathways, such as including protein digestion and absorption, mineral absorption, aminoacyl-tRNA biosynthesis, central carbon metabolism and amino acid metabolism.

### Diversity and Composition of Gut Microbiota

**Figure 7:**
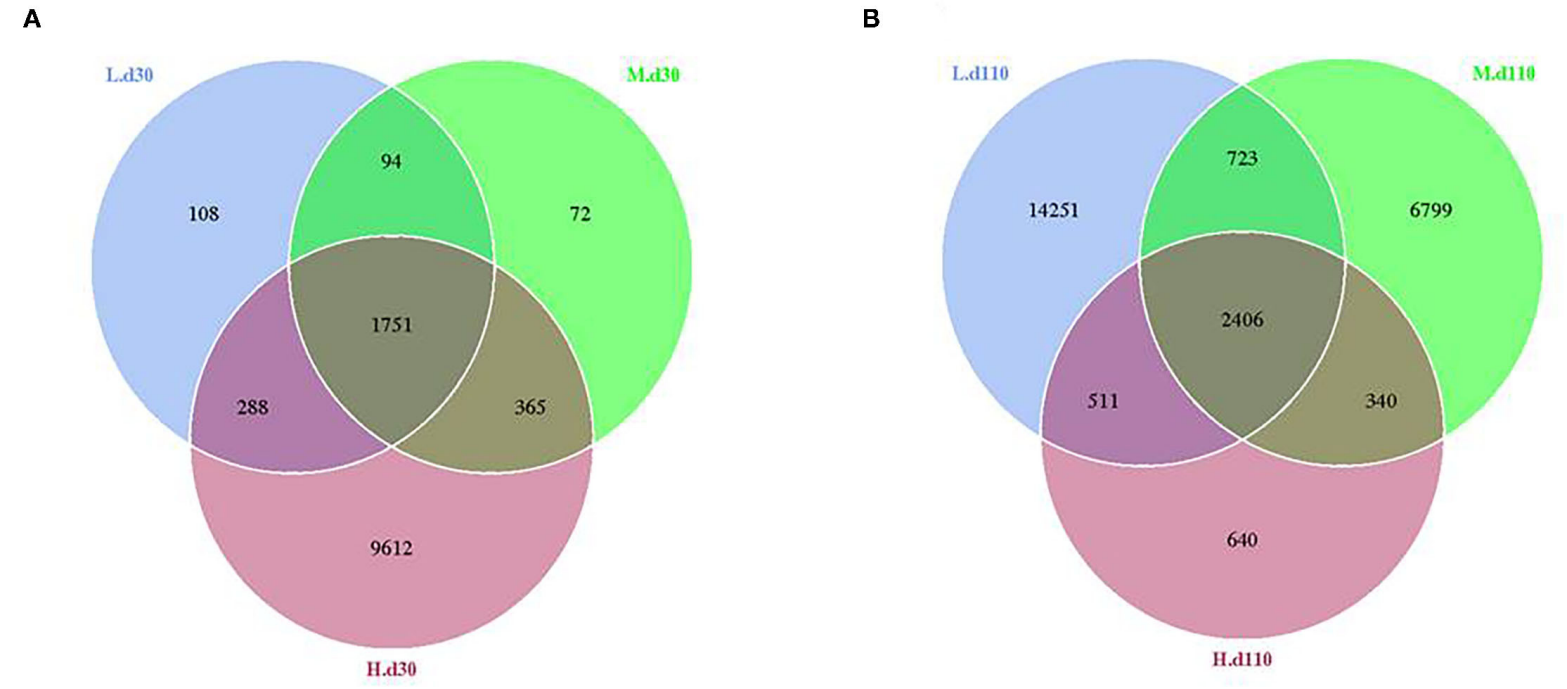
Venn diagram showing the unique and shared OTUs in the different groups at day 30 of gestation **(A)** and day 110 of gestation **(B)**. *N* = 8 for each treatment.

From the Venn analysis of operational taxonomic units (OTUs), 108, 72 and 9,612 unique OTUs on day 30, 14,251, 6,799 and 640 unique OTUs on day 110 were identified in the 1,050, 1,850, and 2,650 mg/kg choline groups, respectively (Figure 7). The alpha diversity index is shown in Table 6; the observed species tended to increase as dietary choline levels increased at day 30 of gestation (*P* = 0.093). However, the observed species (*P* = 0.016), Chao 1 (*P* = 0.014) and ACE (*P* = 0.012) decreased as dietary choline levels increased at day 110 of gestation.

**Table 6:** Differences in bacterial community diversity and richness among the three treatments.

**Item**	**Dietary choline level, mg/kg**			**SEM**	***P*-value**
	**1,050**	**1,850**	**2,650**		
**Day 30**					
Observed species	1,307.1	1,425.9	2,047.7	237.92	0.093
Shannon	6.9	7.3	7.5	0.36	0.566
Chao1	1,634.7	1,758.2	3,535.5	606.70	0.150
ACE	1,673.9	1,819.2	4,014.7	725.88	0.119
**Day 110**					
Observed species	3,214.1	2,307.8	1,631.0	353.55	0.016
Shannon	7.6	7.3	7.8	0.52	0.791
Chao1	6,012.8	4,338.1	1,999.2	864.59	0.014
ACE	6,936.1	4,775.4	2,055.5	1,031.01	0.012

*Data are expressed as least squares means with pooled SEM, N = 8 per treatment*.

**Figure 8:**
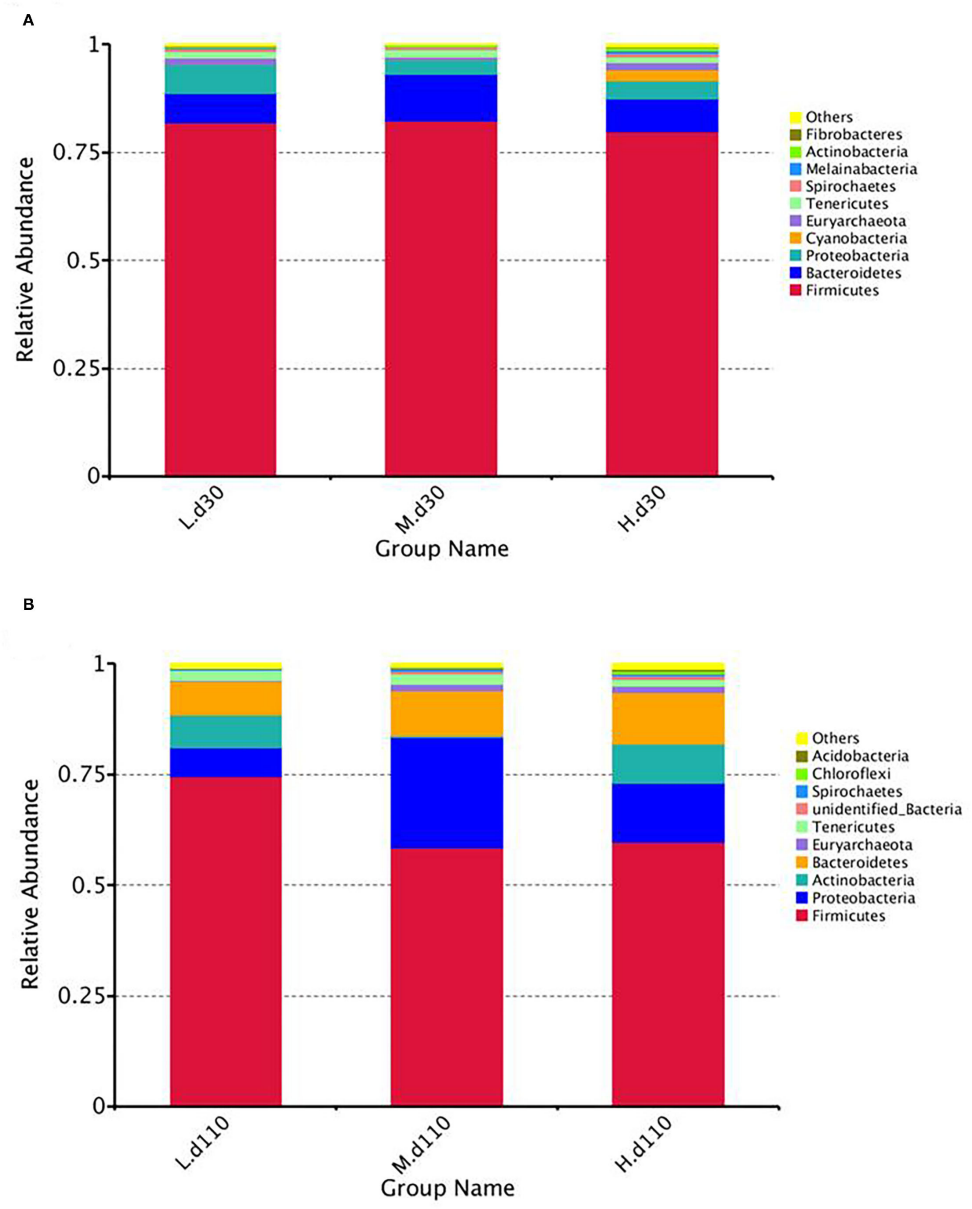
Relative abundance of intestinal microbiota at the phylum levels at day 30 of gestation **(A)** and day 110 of gestation **(B)**. L, 1,050 mg/kg choline group; M, 1,850 mg/kg choline group; H, 2,650 mg/kg choline group. *N* = 8 for each treatment.

At the phylum level, the fecal samples of the sows were dominated by three phyla on both day 30 and day 110: *Proteobacteria, Firmicutes*, and *Bacteroidetes* (Figure 8; Supplementary Table 2). The abundance of *Proteobacteria* (*P* = 0.041) decreased and the abundance of *Actinobacteria* (*P* = 0.040) increased at day 30 of gestation with an increase in dietary choline level (Supplementary Table 2).

**Figure 9:**
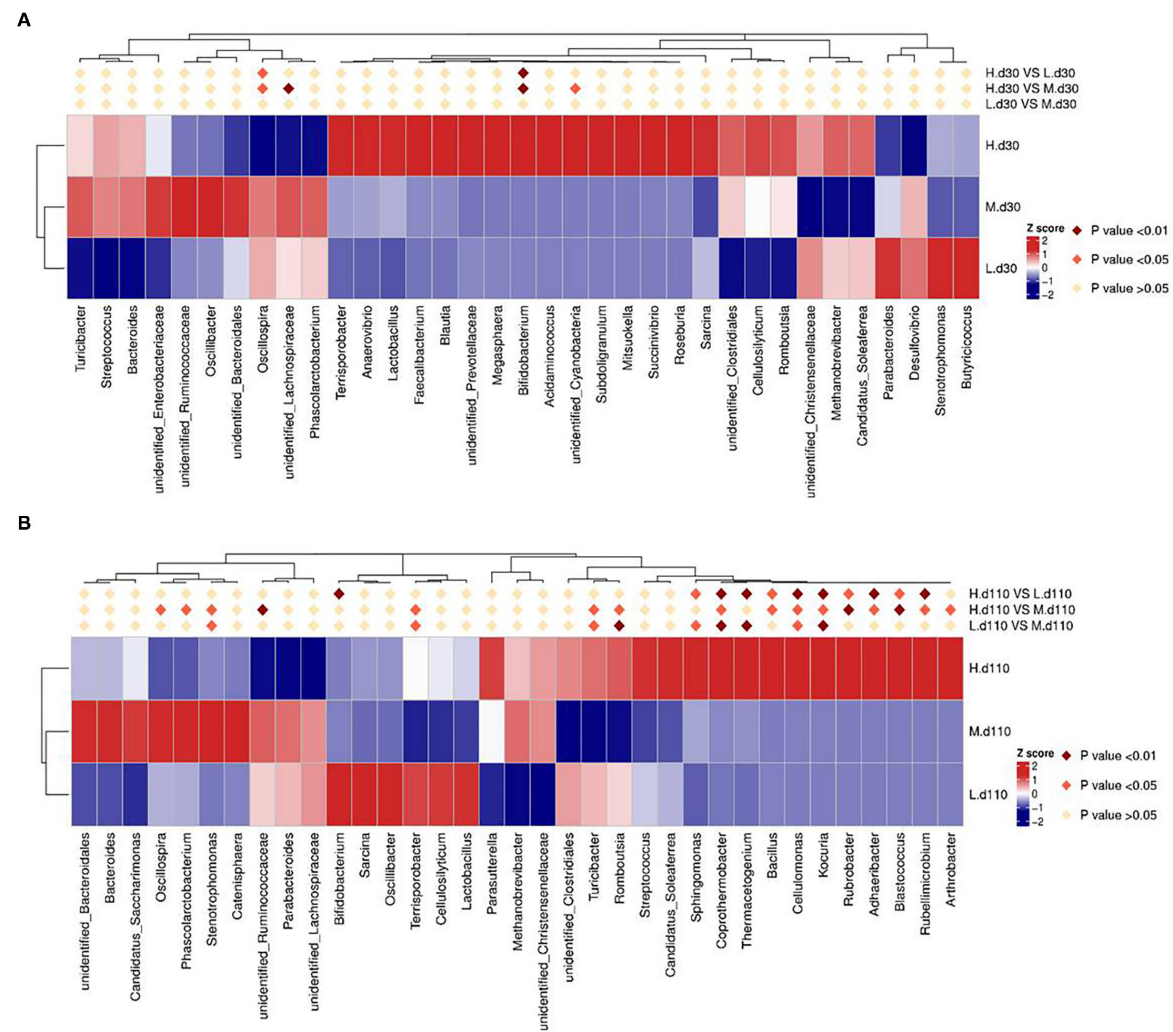
Heat map distribution of the genera with relative abundance (top 30) in sow fecal contents for all treatments at day 30 of gestation **(A)** and at day 110 of gestation **(B)**. Different colors indicate the relative abundance of taxa. L, 1,050 mg/kg choline group; M, 1,850 mg/kg choline group; H, 2,650 mg/kg choline group. *N* = 8 for each treatment.

The relative abundance at the genus level in sow fecal microbiota (top 30) is shown in Figure 9. At day 30 of gestation, the abundance of *Bifidobacterium* in the 2,650 mg/kg choline group was higher than that in the other two groups (*P* < 0.01; Figure 9A), whereas the abundance of *Oscillospira* was lower than that in the other two groups (*P* < 0.05; Figure 9A). Compared with the 1,850 mg/kg choline group, the abundance of *unidentified Lachnospiraceae* in the 2,650 mg/kg choline group significantly decreased (*P* < 0.01; Figure 9A), whereas the abundance of *unidentified Cyanobacteria* significantly increased (*P* < 0.05; Figure 9A). At day 110 of gestation, the abundance of *Coprothermobacter, Bacillus, Cellulomonas, Kocuria, Rubrobacter, Rubellimicrobium, Blastococcus* and *Adhaeribacter* in the 2,650 mg/kg choline group were higher than those in the other two groups (*P* < 0.05; Figure 9B). Compared with the 1,050 mg/kg choline group, the abundance of *Sphingomonas* and *Thermacetogenium* significantly increased (*P* < 0.05; Figure 9B), whereas the abundance of *Bifidobacterium* significantly decreased (*P* < 0.01; Figure 9B) in the 2,650 mg/kg choline group. Compared with the 1,850 mg/kg choline group, the 2,650 mg/kg choline group had a higher abundance of *Terrisporobacter, Turicibacter, Romboutsia* and *Arthrobacter* (*P* < 0.05; Figure 9B), but a lower abundance of *Oscillospira, Phascolarctobacterium, Stenotrophomonas* and *unidentified Ruminococcaceae* (*P* < 0.05; Figure 9B). Compared with the 1,050 mg/kg choline group, the 1,850 mg/kg choline group had a higher abundance of *Kocuria, Cellulomonas, Coprothermobacter, Thermacetogenium, Sphingomonas* and *Stenotrophomonas* (*P* < 0.05; Figure 9B), and a lower abundance of *Terrisporobacter, Turicibacter* and *Romboutsia* (*P* < 0.05; Figure 9B).

## Discussion

The choline requirement for pregnant sows has not been updated since it was established by the NRC in 1979. Modern sows farrow much more piglets than sows in the 1970s; thus, whether the requirement is still suitable for modern sows remains unknown. In addition, the modulatory role of choline on the fetal growth and development are of great interest (7). To the best of our knowledge, the effects of dietary choline on plasma metabolome and gut microbiota of pregnant sows have not been reported to date. Therefore, the major objective of the present study was to investigate the influence of dietary choline levels during gestation on reproductive performance. Specifically, we determined the effects of choline on the plasma metabolome and gut microbiota of gestating sows.

In the current study, the individual birth weight for total piglets born, piglets born alive, and litter weight at weaning increased linearly as dietary choline level increased. In contrast, the within-litter birth weight CV for piglets born alive and suckling piglet mortality decreased linearly. Consistent with our results, studies in animals and humans suggest that maternal choline supplementation during pregnancy has beneficial effects on fetal growth (17, 18). As is generally known, fetal growth is dependent on the placental nutrient supply. A previous study suggested that choline supply can influence placental angiogenic processes via protein kinase C, and the increased placental angiogenesis might contribute to the extraction of more nutrients from the maternal circulation to the fetus (19). Similarly, Kwan et al. (20) reported that maternal choline supplementation improved placental nutrient transporter abundance and nutrient metabolism in mice during late gestation, with subsequent effects on nutrient supply for the developing fetus. In addition, a lower plasma HCY concentration was observed in the 1,850 and 2,650 mg/kg choline groups in comparison with the 1,050 mg/kg choline group in the current study. HCY can be transported into the human placenta through amino acid transporters, which may compete with amino acid transporters, thus impairing amino acid transport (21, 22). In the current study, the decrease in maternal HCY level due to elevated choline intake may decrease its competition with amino acid transporters, thus allowing more amino acids to be transported into the fetus. Consistently, studies in humans have suggested that infant birth weight is negatively correlated with maternal HCY concentration (23–25).

The BF gain of sows increased linearly with an increase in dietary choline levels in our study. This may be associated with the extra choline metabolism in the intestine. A previous study showed that approximately two-thirds of ingested choline could not be absorbed by the intestinal tract, and the unabsorbed choline would be utilized by gut microflora to generate trimethylamine (TMA) (26).TMA is quickly absorbed into the liver by intestinal epithelial cells, where TMA is further metabolized to TMAO (27). Blaak and Canfora (28) demonstrated that circulating TMAO was positively associated with body mass index, body fat composition, and visceral fat mass in overweight and obese individuals without diabetes. Other studies have also shown that TMAO can induce obesity in humans and mice (29, 30). Moreover, the addition of TMAO in the diet of growing-finishing pigs significantly increased the apparent total tract digestibility of fat (31). Therefore, the increased BF gain of sows may be attributed to the increase in dietary fat digestibility and body fat deposition induced by the elevated choline intake, as represented by the increased plasma TMAO concentration in sows.

Dietary choline deficiency can lead to liver injury and inflammation (32). In our study, the plasma from the 1,850 and 2,650 mg/kg choline groups had a lower H_2_O_2_ concentration at day 30 of gestation compared with that of the 1,050 mg/kg choline group. As a major type of reactive oxygen species (ROS), H_2_O_2_ is a molecular culprit that induces lipoperoxidation (14). In addition, in comparison with the 1,050 mg/kg choline group, higher tyramine and dopamine concentrations were detected in the plasma of the 1,850 and 2,650 mg/kg choline groups. A previous study showed that dopamine has a greater antioxidant capacity than vitamin E, and the antioxidant capacity of tyramine was similar to that of vitamin E. Both can effectively inhibit linoleic acid oxidation due to their ROS reduction and scavenging capacity (33). The decreased H_2_O_2_ concentration and the increase in tyramine and dopamine concentrations suggested that the oxidative stress in sows was alleviated by dietary choline supplementation to a certain degree. Studies have shown that the improvement of maternal antioxidant capacity contributes to the reduction of within-litter birth weight variation in neonatal mice or piglets (34, 35). In line with this, the within-litter birth weight CV of born alive piglets decreased as dietary choline concentration increased.

To further understand the underlying mechanisms of dietary choline supplementation on the metabolic status of gestating sows, metabolomic analysis was introduced into the present study. OPLS-DA analyses demonstrated a clear separation of plasma metabolites among these groups, suggesting marked differences in the metabolic profiles due to dietary choline supplementation. A higher concentration of 2-Oxoadipic acid, diethanolamine, L-proline, Leu-Leu, 1-Aminocyclopropanecarboxylic acid, N6, N6, N6-Trimethyl-L-lysine, L-phenylalanine, L-tyrosine and trans-2-Hydroxycinnamic acid were observed in the plasma from the 1,850 and 2,650 mg/kg choline groups compared with the 1,050 mg/kg choline group. L-proline is a precursor for ornithine biosynthesis and can be converted to polyamine by ornithine decarboxylase (36). A recent study reported that maternal dietary L-proline supplementation in Huanjiang mini-gilts increased ornithine decarboxylase protein abundance, polyamine concentration in the fetal intestines, and fetal weight (37). 1-methylhistidine is positively correlated with skeletal muscle injury and the breakdown of muscle proteins (38), suggesting increased choline intake during gestation might reduce maternal protein degradation and improve amino acid metabolic status; thus, more amino acids could be transported from dams to the developing fetus. It is well known that amino acids are the most important precursors for fetal growth and development during gestation (39). In addition, enhanced circulating phenylalanine concentration is usually linked with positive appetite regulation (15). This was consistent with the increased ADFI of sows during lactation induced by increasing dietary choline levels. Vitamin A and cholic acid levels were higher in the 1,850 mg/kg choline group than in the other two groups. Maternal Vitamin A deficiency is also associated with fetal maldevelopment (9). Increased Vitamin A levels might be an important factor contributing to better maternal performance during gestation and lactation (40). Cholic acid, a major primary bile acid, facilitates fat absorption and cholesterol excretion (41). The increased cholic acid concentration indicated that there was better digestion and absorption for sows in the 1,850 mg/kg choline group, which can be explained by the increased BF gain and the higher ADFI during lactation in these sows to some extent. Notably, increased consumption of dietary choline during pregnancy enhanced the concentration of choline metabolism biomarkers. Plasma TMAO concentrations increased as dietary choline levels increased in the current study. It has been shown that elevated plasma TMAO concentrations lead to an increased risk of major adverse cardiovascular disease in humans (42). Furthermore, the plasma of sows in the 1,850 and 2,650 mg/kg choline groups had a higher diethanolamine concentration than that of the 1,050 mg/kg choline group. Diethanolamine is an important endogenous precursor in the synthesis of phospholipids, while excessive diethanolamine treatment may alter phospholipid biosynthesis and disrupt choline homeostasis (43). Collectively, the results showed that choline intake was involved in the core of protein digestion and absorption, as well as vitamin and amino acid metabolism. Thus, an appropriate choline level during gestation could enhance the nutrient supply from the sows to the fetus, but excessive maternal choline intake may lead to adverse effects due to the elevated toxicological metabolites. More studies are needed to clarify the underlying mechanism.

The gut microbiota plays a critical role in nutrient metabolism, immune development, protection from pathogens, and the incidence of many chronic diseases in the host (44). In this study, no influence was found for microbial diversity (Shannon index) among the three choline treatment groups. However, microbial richness of fecal microbiota decreased during late gestation as the dietary choline level increased, as reflected by the decreased community richness indices (observed species, Chao 1 and ACE). Thus, the results indicate that dietary choline level could embellish the gut microbiota of sows.

At the phylum level, *Firmicutes* and *Bacteroidetes* were the most dominant phyla regardless of gestation stage (45), which was in good agreement with previous studies on pigs (45, 46). Remarkably, compared with the 1,050 mg/kg choline group, a lower abundance of *Proteobacteria* was observed at day 110 of gestation in the 1,850 mg/kg choline group. *Proteobacteria* are usually associated with intestinal inflammation, and a large number of bacteria affiliated with this phylum are known to result in gut pathology (47). The 2,650 mg/kg choline group had elevated higher abundances of phylum *Actinobacteria* and genus *Bifidobacterium* than the 1,050 and 1,850 mg/kg choline groups at day 30 of gestation. Although *Actinobacteria* represent only a small percentage, they are pivotal in the maintenance of gut homeostasis (48). A previous study demonstrated that the abundances of the phylum *Actinobacteria* was higher in healthy hosts than in unhealthy hosts with irritable bowel syndrome (49). The genus *Bifidobacterium* is widely used as a probiotic, demonstrating beneficial effects in many pathological conditions (50). On day 110 of gestation, the abundance of genera *Bacillus* and *Cellulomonas* in the 2,650 mg/kg choline group was higher than those in the other two groups. Previous studies have shown that bacteria of the genera *Bacillus* and *Cellulomonas* are included in probiotic preparations for animals, which exert anti-inflammatory effects on the intestinal barrier (51, 52). Moreover, a previous study reported that *Bacillus* in the cecal digesta of mice was positively associated with TMA lyase activity (53). It is well established that TMA lyase plays a key role in converting choline to TMA and then it is oxidized to TMAO; thus, the increased abundance of *Bacillus* is in line with the higher plasma TMAO concentration in the current study. Interestingly, a lower relative abundance of genus *Terrisporobacter* was detected in the 1,850 mg/kg choline group than in the other two groups. *Terrisporobacter*, an anaerobic pathogen (54), has been proven to induce oxidative stress (55). The decrease in the abundance of *Terrisporobacter* was consistent with the lower plasma H_2_O_2_ concentration, which further confirmed the improved oxidative status of sows in the 1,850 mg/kg group. Nevertheless, it is worth noting that the relative abundance of the genus *Bifidobacterium* in the 2,650 mg/kg choline group at day 110 of gestation was significantly lower than that of the 1,050 mg/kg choline group. In addition, the observed species, Chao1 and ACE, decreased with an increase in dietary choline levels. The results indicate that long-term supplementation with high doses of choline might be a potential risk factor for intestinal health. In line with this, a study in mice showed that maternal high-dose choline supplementation induced increased susceptibility to colonic mucosal injury in the pups (56). Thus, these findings suggest that an appropriate dietary choline level would benefit the health of gestating sows and their offspring.

## Conclusion

Taken together, the current results suggest that the dietary choline recommendation of 1,250 mg/kg proposed by NRC (2012) is insufficient for the best reproductive performance of modern hyperprolific sows. Increasing dietary choline levels improved the birth weight of piglets, neonatal piglet uniformity and litter performance during lactation, which could be associated with better antioxidant capability, metabolic status and gut microbiota composition of sows during gestation. However, long-term excessive intake of choline during gestation might elevate the risk of maternal health due to accumulated toxicological metabolites and increased harmful microbes. Further investigations are required to reveal the underlying mechanism of high choline intake on the gut microbiota of gestating sows.

## Data Availability Statement

The raw data supporting the conclusions of this article will be made available by the authors, without undue reservation.

## Ethics Statement

The animal study was reviewed and approved by the Animal Care and Use Committee of the Sichuan Agricultural University. Written informed consent was obtained from the owners for the participation of their animals in this study.

## Author Contributions

WZ, LH, and DW: conceptualization. WZ and LH: data curation. DW: funding acquisition, resources, and validation. WZ, LH, YZha, and ZL: investigation. WZ, LH, YZha, ZL, LC, BF, YL, SX, JL, and ZF: methodology. YZhu: project administration. WZ: software and writing. YZhu, LC, BF, YL, SX, JL, ZF, and DW: supervision. LH and DW: review and editing. All authors contributed to the article and approved the submitted version.

## Funding

This study was supported by National Key R&D Program of China (Grant 2018 YFD0501005), Sichuan Province 135 Breeding Tackle Project (Grant 2016 NYZ0052) and the 111 Project (D17015).

## Conflict of Interest

The authors declare that the research was conducted in the absence of any commercial or financial relationships that could be construed as a potential conflict of interest.

## Publisher's Note

All claims expressed in this article are solely those of the authors and do not necessarily represent those of their affiliated organizations, or those of the publisher, the editors and the reviewers. Any product that may be evaluated in this article, or claim that may be made by its manufacturer, is not guaranteed or endorsed by the publisher.
