# Supplementary material for: Effects of Dietary Choline Levels During Pregnancy on Reproductive Performance, Plasma Metabolome and Gut Microbiota of Sows
Source: Front Vet Sci. 2022 Jan 24;8:771228. doi: 10.3389/fvets.2021.771228 (PMC8818960; doi:10.3389/fvets.2021.771228)
Supplement: Supplementary file 1 [file Data_Sheet_1.docx]

Supplementary Material

# Supplementary Tables

**Supplementary Table 1.** Metabolomics Changes in plasma in 1050 mg/kg choline group, 1850 mg/kg choline group, and 2650 mg/kg choline group

| **Metabolites** | **m/z** | **Rt (s)** | **1850 mg/kg vs 1050 mg/kg** | | | **2650 mg/kg vs 1050 mg/kg** | | | **1850 mg/kg vs 2650 mg/kg** | | |
| --- | --- | --- | --- | --- | --- | --- | --- | --- | --- | --- | --- |
|  |  |  | **VIP** | **Change** | **t-test** | **VIP** | **Change** | **t-test** | **VIP** | **Change** | **t-test** |
| Pro-Val | 214.13 | 323 |  |  |  |  |  |  | 4.80 | ↑ | 0.024 |
| Dihydrouracil | 302.80 | 173 |  |  |  |  |  |  | 2.82 | ↑ | 0.049 |
| 5,6,7,8-tetrahydro-2-Naphthoic Acid | 254.83 | 157 |  |  |  | 1.22 | ↓ | 0.037 | 1.14 | ↑ | 0.007 |
| L-Carnosine | 227.11 | 332 |  |  |  |  |  |  | 1.73 | ↑ | 0.012 |
| L-Arabinono-1,4-lactone | 45.90 | 169 |  |  |  |  |  |  | 1.26 | ↑ | 0.048 |
| Retinol (Vitamin A) | 286.23 | 34 | 2.62 | ↑ | 0.021 |  |  |  | 2.81 | ↑ | 0.016 |
| Cholic acid | 373.27 | 197 | 2.03 | ↑ | 0.020 |  |  |  | 1.05 | ↑ | 0.005 |
| Val-Tyr | 280.14 | 325 |  |  |  |  |  |  | 3.27 | ↑ | 0.034 |
| Diethyltoluamide | 192.14 | 34 |  |  |  | 2.27 | ↓ | 0.007 | 2.66 | ↑ | 0.003 |
| PC (16:0/16:0) | 756.55 | 48 |  |  |  |  |  |  | 5.17 | ↑ | 0.035 |
| Phthalic acid Mono-2-ethylhexyl Ester | 279.16 | 32 | 4.66 | ↑ | 0.082 |  |  |  | 7.58 | ↑ | 0.020 |
| 2-Oxoadipic acid | 348.17 | 141 | 7.59 | ↑ | 0.027 | 1.22 | ↑ | 0.037 | 7.46 | ↑ | 0.050 |
| Diethanolamine | 70.07 | 306 | 3.63 | ↑ | 0.018 | 4.83 | ↑ | 0.004 | 4.38 | ↓ | 0.072 |
| L-Proline | 116.07 | 306 | 5.27 | ↑ | 0.026 | 7.82 | ↑ | 0.003 | 6.35 | ↓ | 0.064 |
| D-Threitol | 290.15 | 181 |  |  |  |  |  |  | 1.00 | ↓ | 0.016 |
| Pantothenate | 220.12 | 264 |  |  |  |  |  |  | 1.18 | ↓ | 0.011 |
| Arg-Ala | 245.15 | 295 |  |  |  | 1.61 | ↑ | 0.015 | 2.04 | ↓ | 0.023 |
| L-Histidine | 373.34 | 154 |  |  |  | 2.07 | ↑ | 0.091 | 2.20 | ↓ | 0.047 |
| Mevalonic acid | 73.48 | 129 |  |  |  | 12.14 | ↑ | 0.079 | 8.93 | ↓ | 0.026 |
| Ergothioneine | 230.09 | 321 | 2.22 | ↓ | 0.046 |  |  |  | 1.94 | ↓ | 0.065 |
| Trimethylamine N-oxide | 76.07 | 320 | 8.29 | ↑ | 0.004 | 12.74 | ↑ | 0.001 | 10.23 | ↓ | 0.036 |
| His-Gly | 213.10 | 135 |  |  |  | 1.02 | ↑ | 0.037 |  |  |  |
| Leu-Leu | 245.18 | 184 | 1.51 | ↑ | 0.003 | 1.32 | ↑ | 0.001 |  |  |  |
| 1-Aminocyclopropanecarboxylic acid | 102.05 | 346 | 1.01 | ↑ | 0.007 | 1.25 | ↑ | 0.000 |  |  |  |
| 3-Methoxy-4-Hydroxyphenylglycol Sulfate | 42.58 | 263 |  |  |  | 1.86 | ↑ | 0.004 |  |  |  |
| L-Tryptophan | 205.10 | 252 |  |  |  | 4.89 | ↑ | 0.045 |  |  |  |
| N6, N6, N6-Trimethyl-L-lysine | 189.16 | 529 | 2.49 | ↑ | 0.005 | 2.07 | ↑ | 0.004 |  |  |  |
| Tyramine | 120.08 | 251 | 8.37 | ↑ | 0.009 | 8.54 | ↑ | 0.004 |  |  |  |
| Guanidoacetic acid | 118.06 | 251 |  |  |  | 1.31 | ↑ | 0.016 |  |  |  |
| Dopamine | 136.07 | 294 | 1.84 | ↑ | 0.043 | 2.14 | ↑ | 0.003 |  |  |  |
| L-Phenylalanine | 166.09 | 251 | 5.46 | ↑ | 0.023 | 6.06 | ↑ | 0.004 |  |  |  |
| L-Tyrosine | 182.08 | 294 | 2.28 | ↑ | 0.060 | 2.75 | ↑ | 0.003 |  |  |  |
| trans-2-Hydroxycinnamic acid | 165.05 | 294 | 1.85 | ↑ | 0.049 | 2.04 | ↑ | 0.006 |  |  |  |
| DL-O-tyrosine | 146.06 | 252 |  |  |  | 1.41 | ↑ | 0.031 |  |  |  |
| DL-Indole-3-lactic acid | 188.07 | 252 |  |  |  | 5.55 | ↑ | 0.048 |  |  |  |
| L-Leucine | 253.00 | 130 |  |  |  | 4.82 | ↑ | 0.022 |  |  |  |
| L-Methionine | 150.06 | 277 |  |  |  | 1.16 | ↑ | 0.042 |  |  |  |
| L-Valine | 289.64 | 116 |  |  |  | 2.13 | ↑ | 0.024 |  |  |  |
| L-Pyroglutamic acid | 130.05 | 363 |  |  |  | 3.50 | ↑ | 0.020 |  |  |  |
| Hypoxanthine | 137.04 | 166 |  |  |  | 5.48 | ↓ | 0.040 |  |  |  |
| 1-Methylhistidine | 170.09 | 384 | 7.00 | ↓ | 0.052 | 7.22 | ↓ | 0.030 |  |  |  |
| Triethanolamine | 150.11 | 165 | 4.85 | ↓ | 0.003 | 5.14 | ↓ | 0.007 |  |  |  |
| N6-methyladenosine | 282.12 | 131 |  |  |  | 1.33 | ↓ | 0.038 |  |  |  |
| His-Ser | 243.11 | 348 | 1.14 | ↑ | 0.047 |  |  |  |  |  |  |
| Indole-2-carboxylic acid | 162.05 | 43 | 2.46 | ↑ | 0.049 |  |  |  |  |  |  |
| Stearic acid | 42.53 | 283 | 7.60 | ↑ | 0.029 |  |  |  |  |  |  |

m/z, mass-to-charge ratio; VIP, variable importance in the projection; Rt, Retention time; Changes with “↑/↓” means increased/decreased after adding choline in diet. “-” means the level of metabolites shows no difference with the control

**Supplementary Table 2.** The relative abundances of nine phyla (%, >1% in at least one sample) and firmicutes/bacteroidetes ratio in 1050 mg/kg choline group, 1850 mg/kg choline group, and 2650 mg/kg choline group

| Item | dietary choline level, mg/kg | | |  |  |
| --- | --- | --- | --- | --- | --- |
|  | 1050 | 1850 | 2650 | SEM | *P*-value |
| d 30 |  |  |  |  |  |
| *Firmicutes* | 82.38 | 82.01 | 79.38 | 3.231 | 0.766 |
| *Proteobacteria* | 5.58^a^ | 2.97^ab^ | 1.76^b^ | 1.107 | 0.041 |
| *Actinobacteria* | 0.28^b^ | 0.28^b^ | 0.80^a^ | 0.169 | 0.040 |
| *Bacteroidetes* | 7.23 | 11.26 | 7.81 | 2.852 | 0.871 |
| *Cyanobacteria* | 0.04 | 0.02 | 0.13 | 0.058 | 0.722 |
| *Euryarchaeota* | 1.41 | 0.73 | 1.73 | 0.671 | 0.910 |
| *Tenericutes* | 1.57 | 1.63 | 1.33 | 0.320 | 0.523 |
| *Spirochaetes* | 0.81 | 0.69 | 0.97 | 0.351 | 0.898 |
| *Melainabacteria* | 0.16 | 0.16 | 0.26 | 0.058 | 0.534 |
| d 110 |  |  |  |  |  |
| *Firmicutes* | 74.21 | 57.83 | 58.89 | 5.240 | 0.099 |
| *Proteobacteria* | 6.70 | 25.28 | 13.86 | 5.201 | 0.062 |
| *Actinobacteria* | 0.50 | 0.47 | 8.72 | 2.010 | 0.054 |
| *Bacteroidetes* | 7.84 | 10.35 | 12.09 | 2.712 | 0.379 |
| *Cyanobacteria* | 0.02 | 0.09 | 0.08 | 0.028 | 0.222 |
| *Euryarchaeota* | 0.27 | 1.38 | 1.26 | 0.518 | 0.062 |
| *Tenericutes* | 2.50 | 2.35 | 1.66 | 0.486 | 0.526 |
| *Spirochaetes* | 0.42 | 0.74 | 0.60 | 0.304 | 0.719 |
| *Melainabacteria* | 0.14 | 0.10 | 0.14 | 0.034 | 0.912 |

**2 Supplementary Figures**

**Supplementary Figure 1**

**(A)**

**
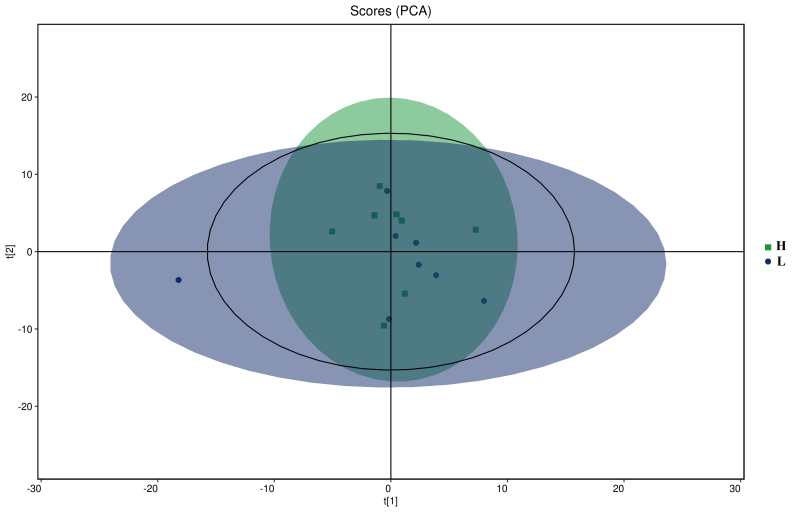
**

**
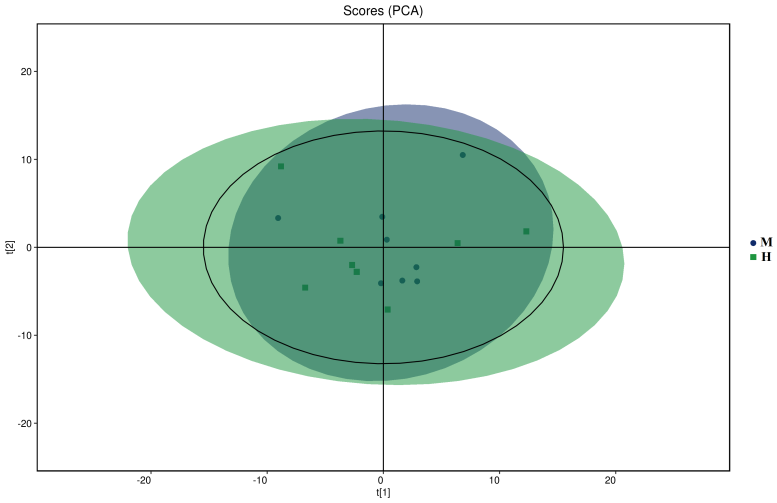
(B)**

**(C)**


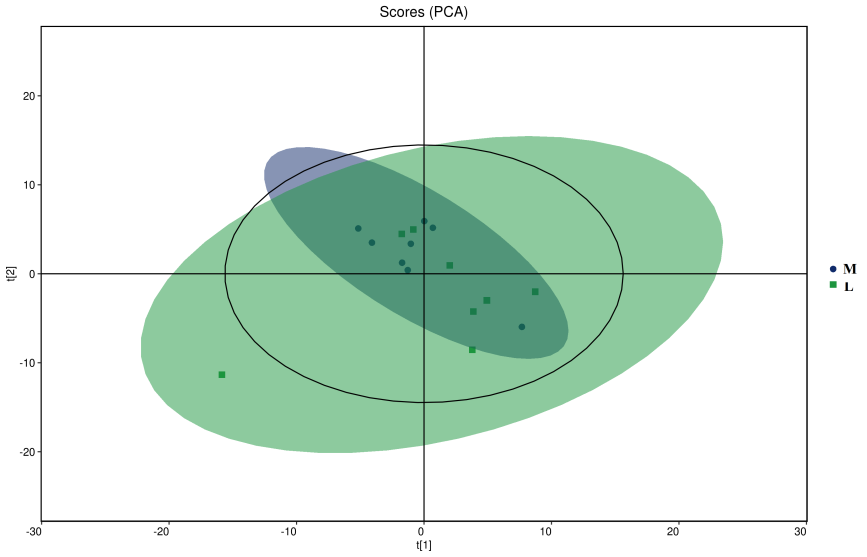


**Supplementary Figure 2**

**(A)**

**
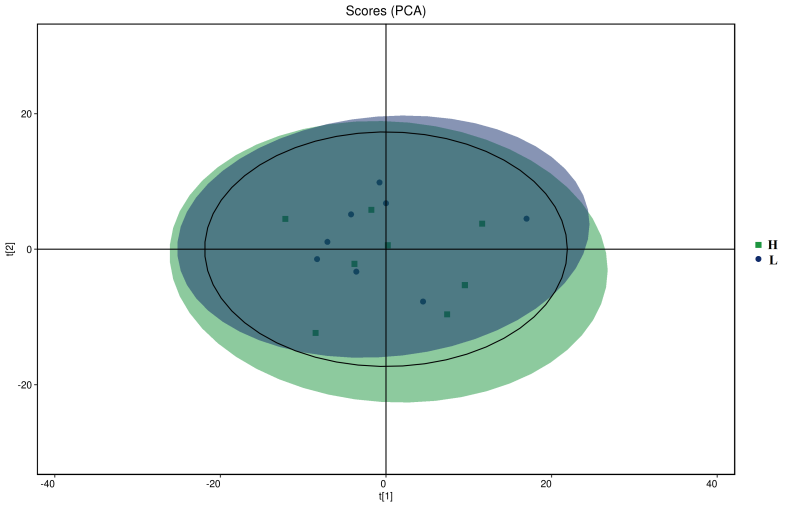
**

**(B)**

**
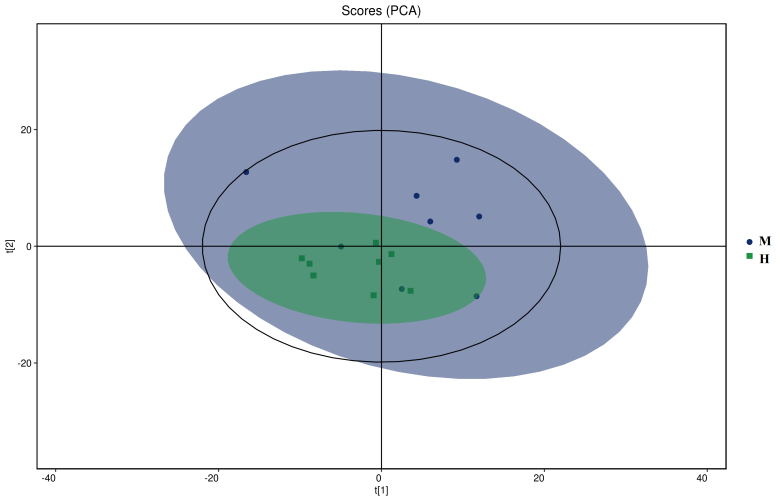
**

**(C)**

**
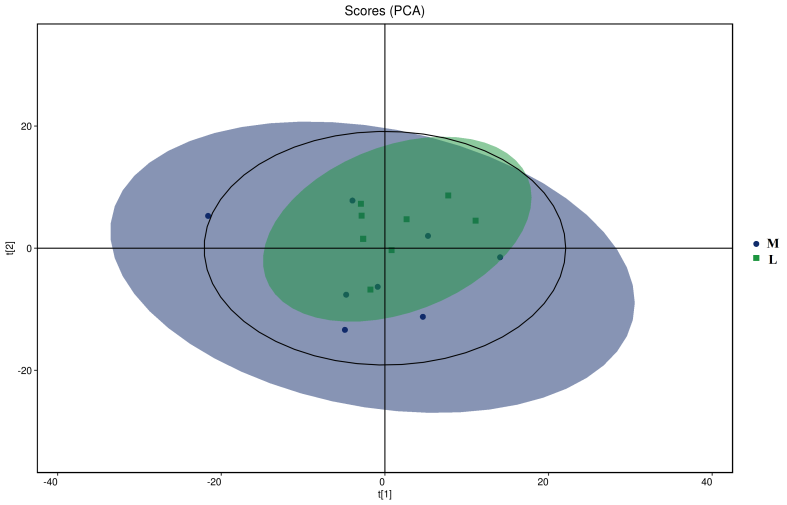
**

**Supplementary Figure 1.** PCA scores plot in ESI+ metabolomics profiles of plasma. t[1] = first principal component. t[2] = second principal component. L = 1050 mg/kg choline group; M = 1850 mg/kg choline group; H = 2650 mg/kg choline group.

**Supplementary Figure 2.** PCA scores plot in ESI^－^metabolomics profiles of plasma. t[1] = first principal component. t[2] = second principal component. L = 1050 mg/kg choline group; M = 1850 mg/kg choline group; H = 2650 mg/kg choline group.
